# Supplementary figures and images for: IgG3 and IgM Identified as Key to SARS-CoV-2 Neutralization in Convalescent Plasma Pools
Source: PLoS One. 2022 Jan 4;17(1):e0262162. doi: 10.1371/journal.pone.0262162 (PMC8726489; doi:10.1371/journal.pone.0262162)

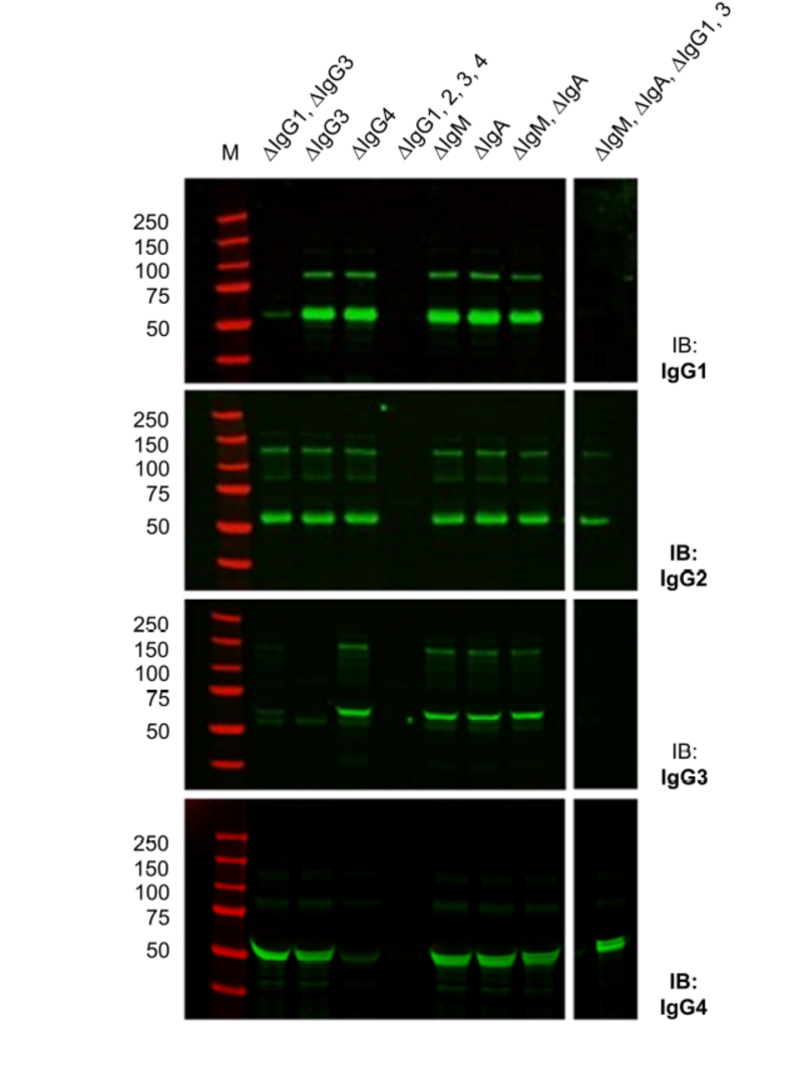

Supplement: S1 Fig — Δ indicates removal of specific Ig classes. IB, immunoblot; M, marker. (TIF) [file pone.0262162.s001.tif]

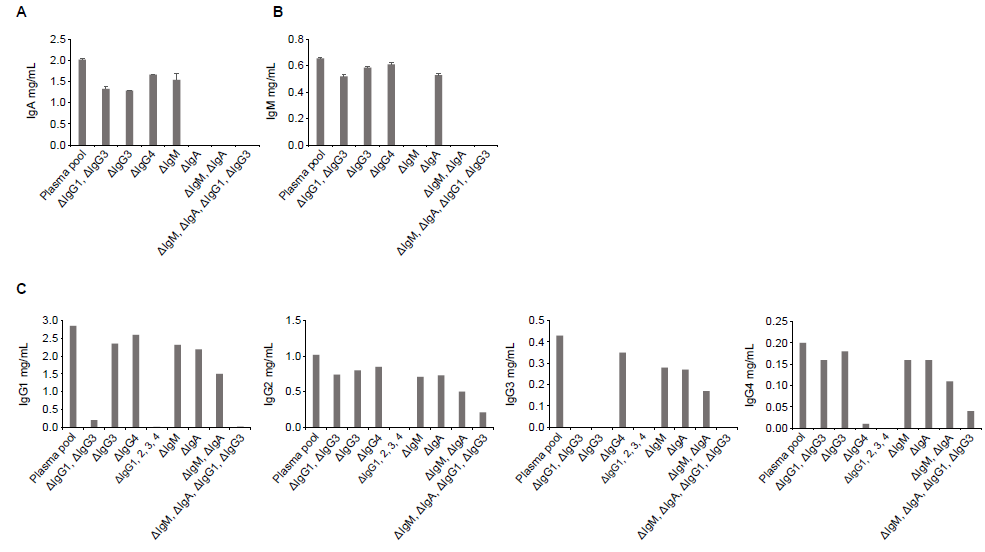

Supplement: S2 Fig — ELISA of Ig classes in depleted plasma pool samples used for SARS-CoV-2 neutralization (A) IgA; (B) IgM; (C) IgG1–4. Bars are mean ± SD. Δ indicates removal of specific Ig classes. (TIF) [file pone.0262162.s002.tif]

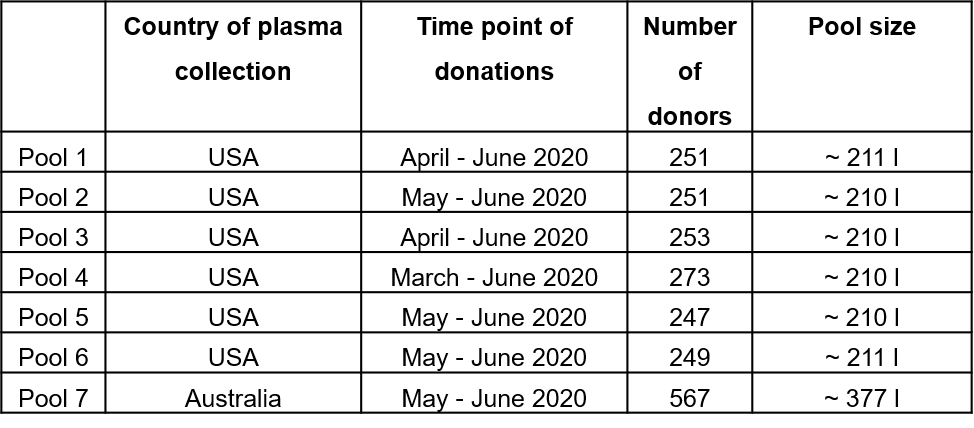

Supplement: S1 Table — (TIF) [file pone.0262162.s003.tif]

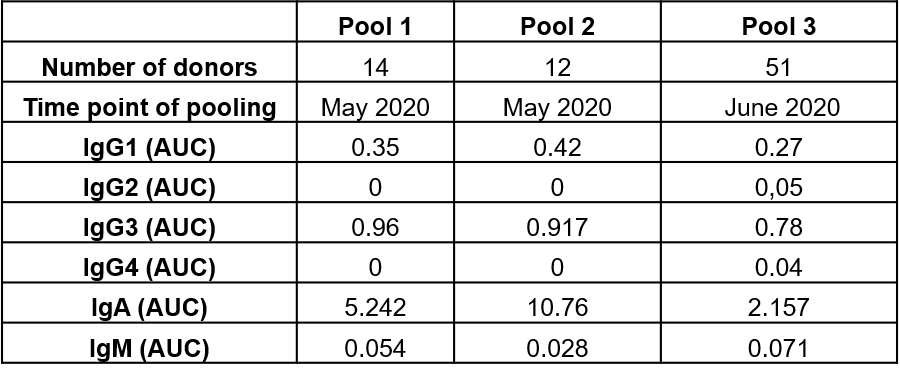

Supplement: S2 Table — (TIF) [file pone.0262162.s004.tif]
