# Supplementary material for: IgG3 and IgM Identified as Key to SARS-CoV-2 Neutralization in Convalescent Plasma Pools
Source: PLoS One. 2022 Jan 4;17(1):e0262162. doi: 10.1371/journal.pone.0262162 (PMC8726489; doi:10.1371/journal.pone.0262162)
Supplement: Attachment — Submitted filename: Response to Reviewers (Rebuttal).docx [file pone.0262162.s005.docx]

| **Editor/Reviewer comment** | **Author response** |
| --- | --- |
| Comment from Dr. Nasrallah (Academic editor) |  |
| Sterlin et al [(Science translational medicine. 2021;13(577)] demonstrate that IgA dominates the early neutralizing antibody response to SARS-CoV-2. In your discussion, could you provide explanation to the differences between your and their findings. | Dear Dr. Nasrallah, we would like to thank you for this valuable comment. We adapted the respective paragraph **(341-354)** in our discussion section as follows: *“It is interesting to note that although we see high levels of IgA binding, this does not correlate with neutralizing activity. This contradicts findings from Chen et al. showing strong correlation between S1-specific and ECD-specific IgA responses and neutralization activity in non-severe patients [33]. However, the neutralization activities of the convalescent sera were shown to significantly decline during the period between 21 days to 28 days after hospital discharge along with a substantial drop in RBD-specific IgA response. These findings are in agreement with Sterlin et al reporting that the early SARS-CoV-2 specific humoral responses were strongly driven by IgA antibodies. In addition, peripheral expansion of IgA plasmablasts was detected shortly after symptom onset and peaked during the third week of the diseases at around day 22 [34]. Serum IgA concentrations decreased notably 1 month after onset of symptoms. These findings demonstrate that IgA may play a more important role in the early phases of infection. The difference with our findings may be due to time points of plasma collection. Our plasma donations were collected from convalescent donors who were symptom free for at least 28 days. It was shown that at this stage (at around day 15-28) IgA and IgG peaked. However, IgA levels start to wane whereas IgG remained stable [35].”* |
| Reviewer 1 |  |
| The manuscript in hand presents results on the potential role of IgM and IgG3, taken from convalescent plasma pools derived from individuals who recovered from the disease, to neutralize the virus in vitro.  The following remarks need your attention: - What is the red line in Fig 1D? couldn’t delineate/differentiate colors in Fig. 1D! | We would like to thank the reviewer for this remark. The red line in Fig 1D (in re-submitted manuscript Fig 2D) is IgG2. As IgG2 (red), IgG4 (purple) and IgM (orange) are at baseline level the lines overlay each other. |
| - In page 4, the 1st paragraph speaks about ALL convalescent pools and how candidates were selected, and their plasma tested. In paragraph 2 of same page the authors discuss six pools in addition to the Australian pool. My assumption was that ALL pools were treated the same way in terms of having to wait for at least 28 days and not only the Australian pool, is this correct? - -  - 1st and 2nd paragraphs of page 4 should be re-written for uniformity. | We would like to thank the reviewer for this comment. The statement that all plasma donations were taken earliest 28 days after positive PCR test is correct. We re-wrote the Patients and specimens chapter in the Material and Methods section of the manuscript (**40-63**) as follows and added some additional information in S1 Table.  *“[…] All individual convalescent plasma donations were tested for NC-binding IgG levels using the high-throughput Abbott Architect System according to the manufacturer’s instructions. Donations above the positive cut off (1.4) were classified as convalescent donations and were used in respective convalescent plasma pools or analyzed directly in the study.*  *Six convalescent plasma pools consisting of 247–273 individual donations were collected by the CSL Plasma donation center network throughout the US. All donations that contributed to the six US plasma pools were collected between March 31, 2020 and June 23, 2020. Fifteen donations were collected in March, 121 in April, 466 in May, and 923 in June 2020. The PCR-positive test date was available for 305 donors, and for these donors, the mean period of convalescent plasma collection after a positive PCR test was 57 +/- 19 days. One convalescent plasma pool with 567 donations was collected by the Australian Red Cross Lifeblood between May 2020 and June 2020 at least 28 days after symptom resolution. Details with respect to the 7 convalescent plasma pools are summarized in S1 Table. […]”*    **Supplemental S1 Table. Overview on analyzed large convalescent plasma pools including pooling time-point, volume, and country of collection.** |
| - The authors used pooled “donations”, were these donations similar to blood bank donations? If so, don’t you think that pooling 200-300 donations into one pool was too much? Such large pooling “system” has the potential to hide any Ig’s variation, why not use smaller number of donations (e.g. 20-50 donations) in each pool for more “realistic” approach? Also,  What is the volume of each pool (i.e. volume range)? and how pooling was done? | We would like to thank the reviewer for this valuable question.  The aim of this study was to establish a profile of antibodies at a broad ‘population level’ in order to maximize the statistical power of the results, which is the key reason for the investigation of large plasma pools in this study.  The comparison of the antibody profiles generated in the seven plasma pools with a set of plasma from individual donors allowed to compare the response between the pools and the individual donors (Fig 3 A-C and Fig 3 D-G).  In addition to individual donors (S1A–D) also convalescent plasma pools with 12-51 donors were analyzed and the data is provided in S2 Table showing comparable results to large plasma pools with IgG3 being the main IgG subclass.    **Supplemental S2 Table:** **Anti-SARS-CoV-2 S1 antibody levels in smaller sized convalescent plasma pools consisting of 12-51 donors**  The following sentence was added to the results section: **(202-204)**. In addition, also smaller plasma pools consisting of 12-51 donors were tested showing comparable results with the large pools (S2 Table)  The question with regard to the pool size/ volume range is now covered in Supplemental S1 Table. The volume range is between 210 l and 377 l.  The procedure of the plasma collection is explained in more detail in the method section (**43-51**). *“Our donors were individuals recovered from a recent illness diagnosed as COVID-19 recruited to provide Source Plasma for further manufacturing into an immune globulin product with high titres of SARS-CoV-2 antibody. At each of their visits at which a sample was provided, a plasmapheresis collection of between 198 and 884 mL was performed. Subjects gave informed consent at the outset of the process for samples of their collection to be tested for various parameters. […]* *Donations above the positive cut off (1.4) were classified as convalescent donations and were used in respective convalescent plasma pools or analyzed directly in the study. ”* |
| - Isn’t there a contradiction between Fig 1C, Fig 1D and Table 1 regarding IgM result outcomes? figure 1C shows background amounts of anti-S1 IgM antibodies which will not make a difference if depleted, whereas Table 1 speaks about significant contribution of IgM to the neutralization process (also line 200)! | We thank the reviewer for this valuable observation and would like  to give the following explanation.  The data shown in figure 1C was generated using commercially available pre-coated ELISA plates from EUROIMMUN coated with Spike S1. The assay is optimized and commercially available for qualitative detection of IgG and the antigen concentration is not further specified for this assay.  We shared the same concern as the reviewer and thus established in collaboration with Siemens Healthineers an ELISA with a defined and optimized concentration of selected SARS-CoV-2 antigens to also detect low levels of IgM in our plasma samples and pools. This data is depicted in Fig 3 and shows higher binding to IgM to S1 and RBD compared to pre-pandemic pools. The respective antigen concentrations were added in the method part (89-90). The results part (**216-223**) was adjusted for clarification.  Early induced IgM appears during acute infection and confers the early immune response. With progressive class-switching to IgG, IgM titers start to decrease. The plasma donations for this study were collected from convalescent donors, who were symptom free for at least 28 days. At this stage low IgM levels are expected.  However, the polyvalent nature of IgM and their avidity towards antigens may be beneficial for neutralisation so that they may exert their inhibtory effect already at low concentration.  The following paragraph was added to the Discussion section **(289-296*)*** *“Collectively, our data indicate that IgM plays a pivotal role in viral neutralization, despite its relatively low abundance. This may be due to the polyvalent nature of IgM and the higher avidity towards antigens compared with divalent IgG and IgA. It has been shown that high avidity of neutralizing antibodies play an important role in humoral response against viral infections. Binding between the receptor-binding domain (RBD) of SARS-CoV-2 and the angiotensin-converting enzyme 2 (ACE2) is of high affinity. In a study from Khatri et al, it was concluded that an efficient neutralization of SARS-CoV-2 would require antibodies of high avidity [15]. High avidity IgM immunoglobulins may thus be capable to exert an inhibitory effect already at low concentration.”* |
| - Lines 235-239: results of Fig 2 (A-C) show minimal or baseline amounts of IgM which contradicts what was exhibited in Fig 2D (specifically the IgM graph). | We would like to thank the reviewer for highlighting this point.  We realized that this confusion may have been caused by not showing the binding data of the individual donors in the same figure as the correlation data.  The correlation data is based on both the seven convalescent plasma pools and selected individual donors showing high, medium and low neutralizing capacity. So we adjusted in the resubmitted manuscript Fig 3 accordingly combining the initial Fig 2 and Supplemental S1 Fig. |
| Reviewer 2 |  |
| 1) Line 38 - Determine if there is an approval in the bioethics committee at this stage of the methodology. This was not commented in the manuscript. | We would like to thank the reviewer for this remark.  There is no additional approval in the bioethics committee needed. We added the following statement to the method part **(43-47)**: “*Our donors were individuals recovered from a recent illness diagnosed as COVID-19 recruited to provide Source Plasma for further manufacturing into an immune globulin product with high titres of SARS-CoV-2 antibody. At each of their visits at which a sample was provided, a plasmapheresis collection of between 198 and 884 mL was performed. Subjects gave informed consent at the outset of the process for samples of their collection to be tested for various parameters”*. |
| 2) Line 42 and others - In my way of understanding all the supplementary figures could be placed as the main ones. These figures contain a lot of relevant information fundamental to the consistency of the article. | We would like to thank the reviewer for this suggestion. Figure S1 was put into Fig 3 in the main text and re-named to Fig 3 D-G. Figure S2 was added to the method section as Fig 1. The WB and ELISA data of the depletion experiment remain as Suppl. Figs 1+2. |
| 3) Line 103 - to be more didactic about the presentation of antibody depletion, I find it interesting to put the scheme together with the methodology. | We would like to thank for this suggestion and added the scheme showing the depletion workflow to the method part as Fig 1. |
| 4) Line 142 – insert the reference of this methodology used by the group. | The method part was adjusted as follows **(152-153)**: *“Ig classes in plasma pools used for depletion experiments were quantified using a nephelometric method according to manufacturers instructions (Siemens Healthineers)”*. |
| 5) Line 155 - add reference too | The following references were added **(164)**  *Spearman C. The method of ‘right and wrong cases’ (‘Constant stimuli’) without Gauss’s formulae. Br J Psychology 1904-1920. 1908;2: 227–242. doi:10.1111/j.2044-8295.1908.tb00176.x*  *Kärber G. Beitrag zur kollektiven Behandlung pharmakologischer Reihenversuche. Naunyn-schmiedebergs Archiv Für Exp Pathologie Und Pharmakologie. 1931;162: 480–483. doi:10.1007/bf01863914* |
| 6) Line 267 - Conversely, do the authors have any postulation or comment regarding the low efficiency of neutralization in IgG2 and IgG4? | We thank the reviewer for raising this question. A short paragraph discussing the roles of Ig subclasses about their neutralization and binding efficiency has been added to the Discussion section along with citations of recent work from other groups **(326-339).**  *“IgG2 and IgG4, the other subclasses of IgG did not contribute to either the binding of the tested SARS-CoV-2 antigens or the neutralization of the virus similar to the observation in other studies [17,28,29]. A recent study showed that IgG4 in fact is a marker for mortality with patients having higher anti-RBD IgG4 levels dying during 8-14 and 15-21 days [30]. This suggests that the antibody subclass elicitation to SARS-CoV-2 is broad and correlates strongly to the characteristics of the donor population and sample collection time. Additionally, the IgG subclasses also have variable binding and neutralizing capabilities against protein antigens [31]. IgG1 and IgG3 responses are mostly generated against soluble and membrane protein antigens whereas IgG2 and IgG4 arise against bacterial capsular polysaccharide and allergen molecules respectively. Interestingly, it has been shown that expressing the same spike protein binding monoclonal antibody in human IgG1-4 background resulted in 5-fold superior binding affinity and 50-fold superior neutralization capacity in case of IgG3 over other subclasses. The authors postulate that IgG3 elicits a superior binding and neutralization effect against SARS-CoV-2 in an avidity-dependent manner via cross-linking the spike protein on the viral surface [32]. ”* |
| 6. PLOS authors have the option to publish the peer review history of their article (what does this mean?). If published, this will include your full peer review and any attached files. | We would like to have the peer review history published. |
