# Supplementary material for: IgG3 and IgM Identified as Key to SARS-CoV-2 Neutralization in Convalescent Plasma Pools
Source: PLoS One. 2022 Jan 4;17(1):e0262162. doi: 10.1371/journal.pone.0262162 (PMC8726489; doi:10.1371/journal.pone.0262162)
Supplement: Attachment — Submitted filename: Rebuttal letter_Plos One_20211215.docx [file pone.0262162.s006.docx]

Reviewer #1: Since the last review, the authors have adequately addressed issues raised by the reviewer. My remaining issue is the IgA! In their rebuttal, the authors attached a table (Supplemental S2 Table: Anti-SARS-CoV-2 S1 antibody levels in smaller sized convalescent plasma pools consisting of 12-51 donors); the table clearly shows the dominance of IgA, as measured by AUC, as neutralizing Ab, although the authors still maintain that IgA is of less importance in this regard after 28 days! The pooled plasma used in the study is about 28 days post infection, and I understand that IgA was shown, in previous studies, of less importance and declines around a month post infection, your data says, in several places a different outcome! The question remains: Don't you think that IgA should still be considered as a valid option? The rationale for conducting the study is to justify, in the future, using IgG3 and IgM as passive therapeutic Abs. Don't you think that IgA would be a more valid candidate over IgG's and IgM especially in severe lung disease since it is a secretory Ab?

We thank the reviewer for the critical observation. Indeed, we were also surprised by the high binding activity of IgA. However, we would like to point out that although the Table S2 and Fig. 2C, D show a high AUC value for IgA (both in large and small plasma pools), it reflects only Anti-S1-binding activity and not neutralization of the SARS-CoV-2 virus. In our study, we have linked antigen-binding and ascertained relative contribution of each Ig classes towards neutralizing activity by two methods:

1. correlation analysis between neutralization and antigen binding

2. experimentally via Ig-class depletion

First, we found only a moderate correlation between IgA antigen-binding and neutralization activity with correlation coefficients IgG3>IgM>IgG1>IgA. Second, we found with our depletion study again the same trend of contribution towards neutralization with IgG3>IgM>IgG1>IgA. These data unambiguously show that among the major Ig classes of interest, IgA to be of least neutralizing potency in the tested samples. Additionally, the plasma samples used in the study were from convalescent individuals at >28 days post-recovery phase where IgA levels have been shown to wane (Sterlin et al.). A longitudinal examination of SARS-CoV-2 neutralizing IgA and IgG levels can provide definitive answers but this was not in the purview of the current study.

We however realize that for a respiratory infectious disease, especially the secreted form of IgA could be a means of early defense against pathogens and therefore a potential therapeutic option. But secretory IgA predominant in mucosal tissue is dimeric and was shown to have different neutralizing activity from the monomeric plasma IgA (Sterlin et al.). Since, we have not tested the secretory form of IgA in this study, we cannot comment on that.

As our aim was to create a passive immunotherapy for IV application, we focused on plasma samples which clearly indicated IgG3 and IgM to be the prime drivers of neutralization in plasma. This of course does not rule out mucosal administration of IgA to prevent infection with SARS-CoV-2 but to draw this conclusion from our data would be speculative at best.

To make this point clearer, we have added the following sentences in the manuscript in sentence 341

„ *Interestingly, the high binding levels of IgA do not translate to a strong neutralizing activity. We rather see a moderate level of correlation between antigen-binding and neutralization activity of IgA compared to IgG3 and IgM in the tested samples. In fact, similar correlation coefficients were observed for IgA and IgG1. The Ig-depletion study also indicated presence of low to moderate levels of neutralization contribution by IgA.* “
